# Role of alternative splicing signatures in the prognosis of glioblastoma

**DOI:** 10.1002/cam4.2666

**Published:** 2019-11-01

**Authors:** Zu‐cheng Xie, Hua‐yu Wu, Yi‐wu Dang, Gang Chen

**Affiliations:** ^1^ Department of Pathology The First Affiliated Hospital of Guangxi Medical University Nanning Guangxi Zhuang Autonomous Region P. R. China; ^2^ Department of Cell Biology and Genetics School of Pre‐clinical Medicine Guangxi Medical University Nanning Guangxi Zhuang Autonomous Region P. R. China

**Keywords:** alternative splicing, glioblastoma, prognosis, prognostic signature, TCGA

## Abstract

**Background:**

Increasing evidence has validated the crucial role of alternative splicing (AS) in tumors. However, comprehensive investigations on the entirety of AS and their clinical value in glioblastoma (GBM) are lacking.

**Methods:**

The AS profiles and clinical survival data related to GBM were obtained from The Cancer Genome Atlas database. Univariate and multivariate Cox regression analyses were performed to identify survival‐associated AS events. A risk score was calculated, and prognostic signatures were constructed using seven different types of independent prognostic AS events, respectively. The Kaplan‐Meier estimator was used to display the survival of GBM patients. The receiver operating characteristic curve was applied to compare the predictive efficacy of each prognostic signature. Enrichment analysis and protein interactive networks were conducted using the gene symbols of the AS events to investigate important processes in GBM. A splicing network between splicing factors and AS events was constructed to display the potential regulatory mechanism in GBM.

**Results:**

A total of 2355 survival‐associated AS events were identified. The splicing prognostic model revealed that patients in the high‐risk group have worse survival rates than those in the low‐risk group. The predictive efficacy of each prognostic model showed satisfactory performance; among these, the Alternate Terminator (AT) model showed the best performance at an area under the curve (AUC) of 0.906. Enrichment analysis uncovered that autophagy was the most enriched process of prognostic AS gene symbols in GBM. The protein network revealed that UBC, VHL, KCTD7, FBXL19, RNF7, and UBE2N were the core genes in GBM. The splicing network showed complex regulatory correlations, among which ELAVL2 and SYNE1_AT_78181 were the most correlated (*r* = −.506).

**Conclusions:**

Applying the prognostic signatures constructed by independent AS events shows promise for predicting the survival of GBM patients. A splicing regulatory network might be the potential splicing mechanism in GBM.

## Graphical abstract

Applying the prognostic signatures constructed by independent alternative splicing events shows promise for predicting the survival of glioblastoma (GBM) patients. A splicing regulatory network might be the potential splicing mechanism in GBM.

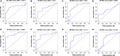

## 1 INTRODUCTION

Glioblastoma (GBM) remains the most aggressive and malignant form of primary central nervous system tumor in adults. Glioblastoma is defined as a grade IV tumor characterized by high heterogeneity and a dismal prognosis, according to the World Health Organization guidelines.^1^ The current first‐line treatment for newly diagnosed GBM is maximal safe resection followed by chemoradiation.^2^, ^3^, ^4^, ^5^, ^6^ Despite aggressive interventions, the disease almost inevitably turns into recurrent GBM, for which no standard and effective treatment approach has been shown to prolong patient survival significantly.^7^, ^8^, ^9^, ^10^, ^11^, ^12^ The overall survival of recurrent GBM is generally no more than half a year.^13^, ^14^, ^15^, ^16^ Consequently, incurable GBM exerts challenging pressure on future work related to searching for novel treatment targets.

Alternative splicing (AS) is the primary driving force for generating diverse proteins, which is the basis for the remarkable and complex functional regulation seen in eukaryotic cells. Alternative splicing is a nearly ubiquitous process, occurring in about 95% transcripts.^17^ Research over the past few decades has provided plenty of evidence concerning the role of AS in cancer. The aberrations of AS in cancers primarily include four categories: (a) AS alterations in oncogenes or tumor suppressor genes; (b) splicing factors mutations; (c) alterations in the upstream signaling pathways that decontrol splicing factors; and (d) aberrations in cancer‐specific spliceosomal components.^18^ Targeting aberrant splicing has provided novel perspectives for clinical therapy strategies.^19^, ^20^ Research on AS in GBM is continually emerging, increasing understanding about the role of AS in GBM. For example, Barbagallo et al found that circSMARCA5 negatively regulated splicing of VEGFA through splicing factor SRSF1, which exerted antiangiogenic function.^21^ Mogilevsky et al discovered that the manipulation of MKNK2 AS significantly suppressed the oncogenic properties of GBM cells and resensitized the cells to chemotherapy, which suggested a novel treatment strategy for clinical practice.^22^ However, the literature primarily focuses on one AS and is lacking an exhaustive overview of all splicing events. In Sadeque's study, the prognostic value of alternative exon usage, a broad category of AS, was comprehensively investigated in GBM patients. Over 2400 alternative exon usage associated prognostic genes in GBM were identified, which provided great inspiration in mining prognostic biomarkers for GBM patients.^23^ Nevertheless, constructing a survival prediction model and investigating the potential AS regulatory mechanism, which are important for both clinical practice and AS research, are lacking in Sadeque's study. Therefore, conducting a study to explore prognostic splicing models and possible AS regulatory mechanism in GBM is necessary.

The TCGA program provides a vast resource of genomic, epigenomic, transcriptomic, and proteomic data related to 33 different cancers.^24^ Well‐documented and freely accessible data make the analysis of AS in cancers possible. Many studies have used TCGA splicing data to investigate AS events and their clinical value related to cancers, such as lung cancer,^25^ bladder cancer,^26^ prostate cancer,^27^ ovarian cancer,^28^ and gastrointestinal adenocarcinomas.^29^ However, no study has comprehensively investigated AS events and their prognostic value related to GBM.

Consequently, we aimed to identify survival‐associated AS events and construct prognostic signatures to predict the survival of GBM patients using the splicing data of 155 samples in TCGA database. We also intended to investigate the possible regulatory mechanism of AS in GBM by constructing an interesting splicing network between AS events and splicing factors. This is the first study to systematically identify survival‐associated AS events and to use splicing signatures for the prediction of survival of GBM patients . We hope this study will help scholars understand the spliceosome and provide new perspectives related to GBM‐related clinical practice.

## 2 MATERIALS AND METHODS

### 2.1 Collecting AS events and GBM‐related clinical data

The splicing profiles of seven types of AS, including alternate acceptor site (AA), alternate donor site (AD), alternate promoter (AP), alternate terminator (AT), exon skip (ES), mutually exclusive exons (ME), and retained intron (RI) were freely available in the TCGA database. TCGASpliceSeq (http://bioinformatics.mdanderson.org/TCGASpliceSeq), is a resource for investigation of mRNA AS patterns for 33 different tumor types in TCGA database, was used for downloading the splicing profiles of GBM.^30^ The profile of the AS events was defined by a percent‐spliced‐in (PSI) value, which ranged from zero to one. Each AS event was presented as a combination of the gene symbol, splicing type, and splicing ID number. The clinical survival data of GBM patients were also downloaded from the TCGA database. Only those patients with an overall survival over 90 days were selected for the purpose of excluding the patient whose death is not because of tumor itself, but other factors such as surgical complications.

### 2.2 Survival analysis and prognostic signatures for AS events

The AS events and survival information of GBM patients were matched using TCGA ID. For each type of AS event, univariate Cox regression analysis was achieved to screen prognostic AS events (*P* < .05). The most significant AS events, namely for AA (*P* < .001), AD (*P* < .005), AP (*P* < .0005), AT (*P* < .001), ES (*P* < .0005), ME (*P* < .05), RI (*P* < .005) were collected. Moreover, we took seven types of AS events as a whole, namely seven combined‐AS, to select the most significant AS events (*P* < .0001) for further analysis. Multivariate Cox regression was then applied to identify the independent prognostic AS events (*P* < .05). The prognostic signatures for each type of AS were constructed using the significantly independent AS events. A risk score for each splicing prognostic signature was achieved using the formula: risk score = $\sum_{i}^{n}PSIi\times \beta i$, where *β* was the regression coefficient in multivariate Cox regression.^26^, ^27^ The median value of the risk score was employed as a threshold value to divide the patients into high‐risk and low‐risk groups. A Kaplan‐Meier curve was plotted to determine the survival rate of GBM patients in the high‐ and low‐risk groups. The 3‐year survival rate of GBM patients was predicted in each splicing model using the receiver operator characteristic (ROC) curve. To compare the predictive efficacy of each prognostic signature in GBM, a survival package was applied to calculate the estimated area under the curve (AUC) of the ROC curve.^26^, ^29^, ^31^, ^32^, ^33^

### 2.3 Construction of the Upset plot and gene enrichment analysis

Because one gene may undergo multiple splicing, we wondered about the details concerning genes and splicing in GBM. As a result, we applied the UpsetR package to draw an Upset plot, which makes intersections in interactive sets more lucid. To acquire biologically enriched pathways, we compiled the top significant gene symbols of the prognostic AS events. The ClusterProfiles package in R software 34 was used to obtain the functionally enriched terms of genes (*P* < .05) in GBM. A *P* value and a *q* value both below .05 was regarded as significant. The top 20 significantly enriched terms were displayed as a bar plot and dot plot. A protein‐protein interactive network of the gene assembles (*P* < .01) was achieved using the STRING database (https://string-db.org/).^35^ A confidence score over 0.9 was used to identify the most confident interactions.

### 2.4 Differentially expressed splicing factors and the construction of a splicing network

The splicing factor is an important regulator in the AS process. Consequently, we gathered the human splicing factors in the SlpiceAid2 database (http://www.introni.it/spliceaid.html).^36^ The mRNA profile data of the splicing factors in both GBM and normal tissues were obtained from the TCGA database. The Deseq 2 package 37 in R software was adopted to screen for differentially expressed splicing factors. A fold change value over 2 and below 0.5 was regarded as up‐regulated and down‐regulated, respectively. A *P* value less than .05 was regarded as statistically significant. Besides, the prognostic value of the differentially expressed splicing factors was also evaluated using Kaplan‐Meier survival curve. Afterwards, the correlation between differentially expressed splicing factors and independent AS events was investigated. Spearman correlation analysis was utilized using SPSS version 25.0 (SPSS, Inc). Significant correlation (*P* < .05) was visualized as an interactive network using Cytoscape version 3.71.^38^

## 3 RESULTS

### 3.1 Details of AS events

**Figure 1:**
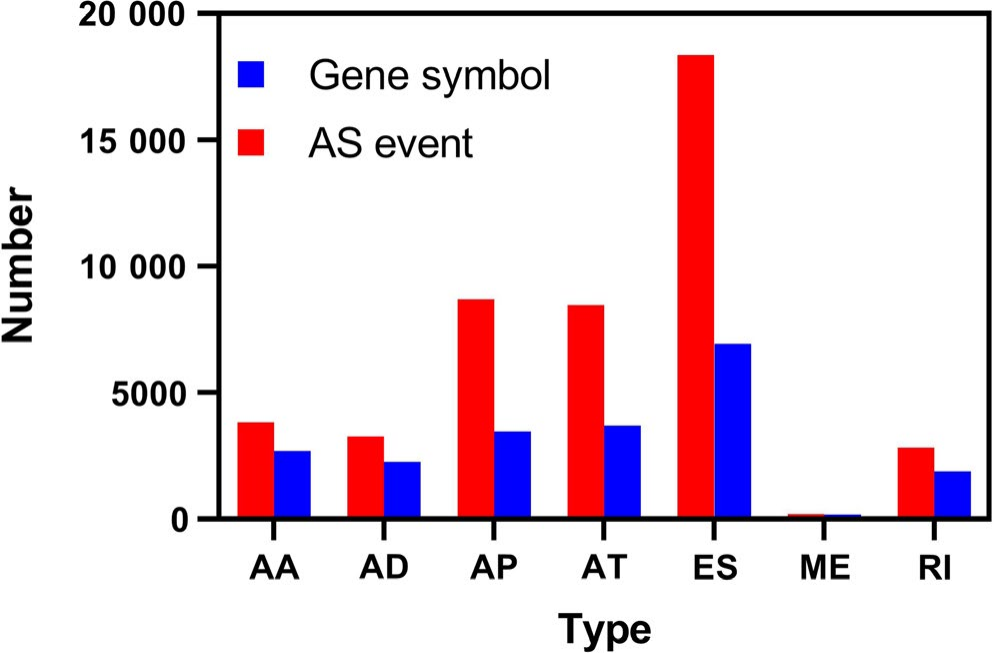
A bar diagram displaying the number of alternative splicing events and gene symbols. AA, alternate acceptor site; AD, alternate donor site; AP, alternate promoter; AT, alternate terminator; ES, exon skip; ME, mutually exclusive exons; RI, retained intron

In summary, 134 GBM patients with 45 610 AS events detected in 10 433 gene symbols were collected. These results comprised 3827 AAs in 2684 genes, 3269 ADs in 2270 genes, 8686 APs in 3476 genes, 8456 ATs 3695 genes, 18 360 ESs in 6934 genes, 184 MEs in 180 genes, and 2828 RIs in 1897 genes (Figure 1). Exon skip was the most dominant splicing type, followed by the AP splicing type. The number of AS events is larger than the total number of genes, implying that one single gene may have undergone multiple splicing.

### 3.2 Survival‐associated AS events

A total of 134 GBM samples with overall patient survival over 90 days were included in the univariate Cox regression analysis. In the univariate Cox regression, 2355 survival‐associated AS events, including 174 AAs, 160 ADs, 514 APs, 404 ATs, 942 ESs, 10 MEs, and 141 RIs, were found. The most significant AS events—6 AAs, 24 ADs, 11 APs, 9 ATs, 17 ESs, 10 MEs, 21 RIs, and 14 seven combined events—were chosen for multivariate Cox regression. Finally, independent prognostic AS events, including 5 AAs, 5 ADs, 5 APs, 5 ATs, 8 ESs, 5 MEs, 8 RIs, and 7 seven combined events, were chosen to construct prognostic signatures. These independent survival‐associated AS events are promising to act as prognostic biomarkers and therapy targets.

**Figure 2:**
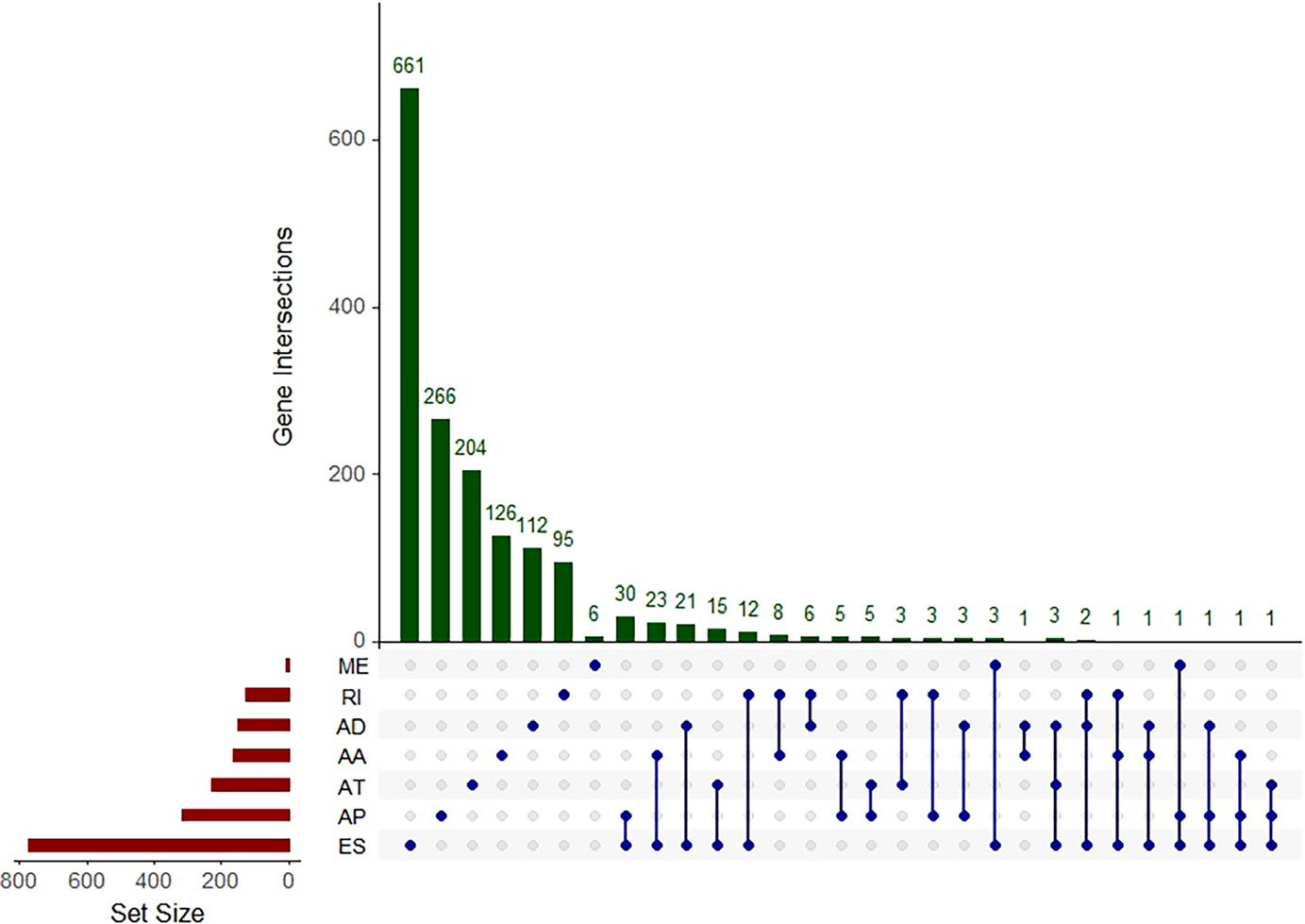
An Upset plot displaying the intersections of different types of alternative splicing events. AA, alternate acceptor site; AD, alternate donor site; AP, alternate promoter; AT, alternate terminator; ES, exon skip; ME, mutually exclusive exons; RI, retained intron

In order to display the overview of splicing, we visualized the intersecting sets of the seven types of survival‐associated AS events. As shown in Figure 2, one gene may have up to three types of AS events. For example, COPZ1, YY1AP1, NFYC, PLEKHM1, TMUB2, ISCU, CHCHD3, ANKS3, VEZT, FYN, and CEP63 all had three types of AS events; ES events existed in all genes.

### 3.3 Enrichment analysis and protein network

**Figure 3:**
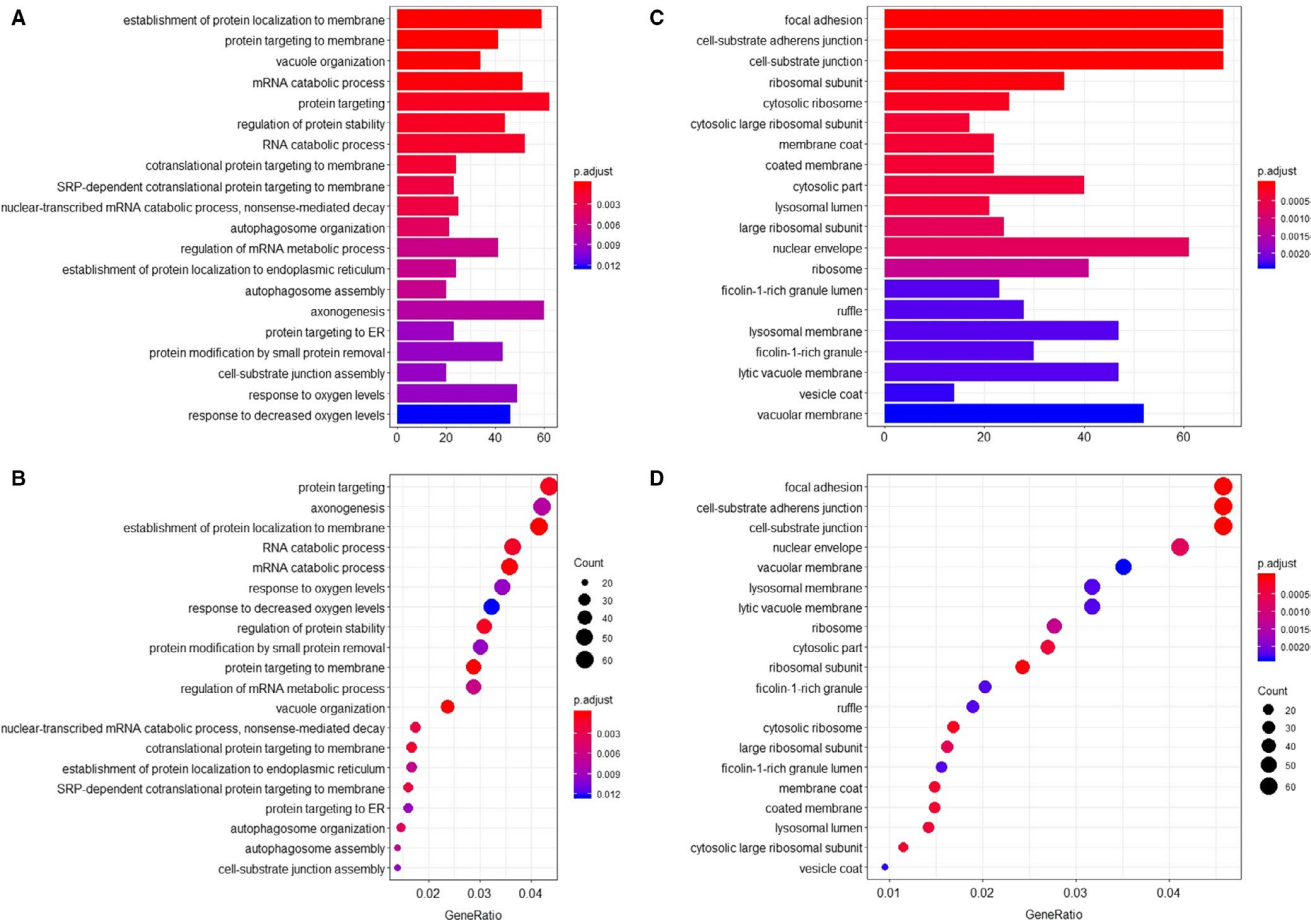
The enrichment analysis of gene symbols in survival‐associated alternative splicing events. The top 20 enriched terms are displayed. A, A bar chart showing the terms of biological process. B, A bubble chart showing the terms of biological process. C, A bar chart showing the terms of cellular component. D, A bubble chart showing the terms of cellular component

**Figure 4:**
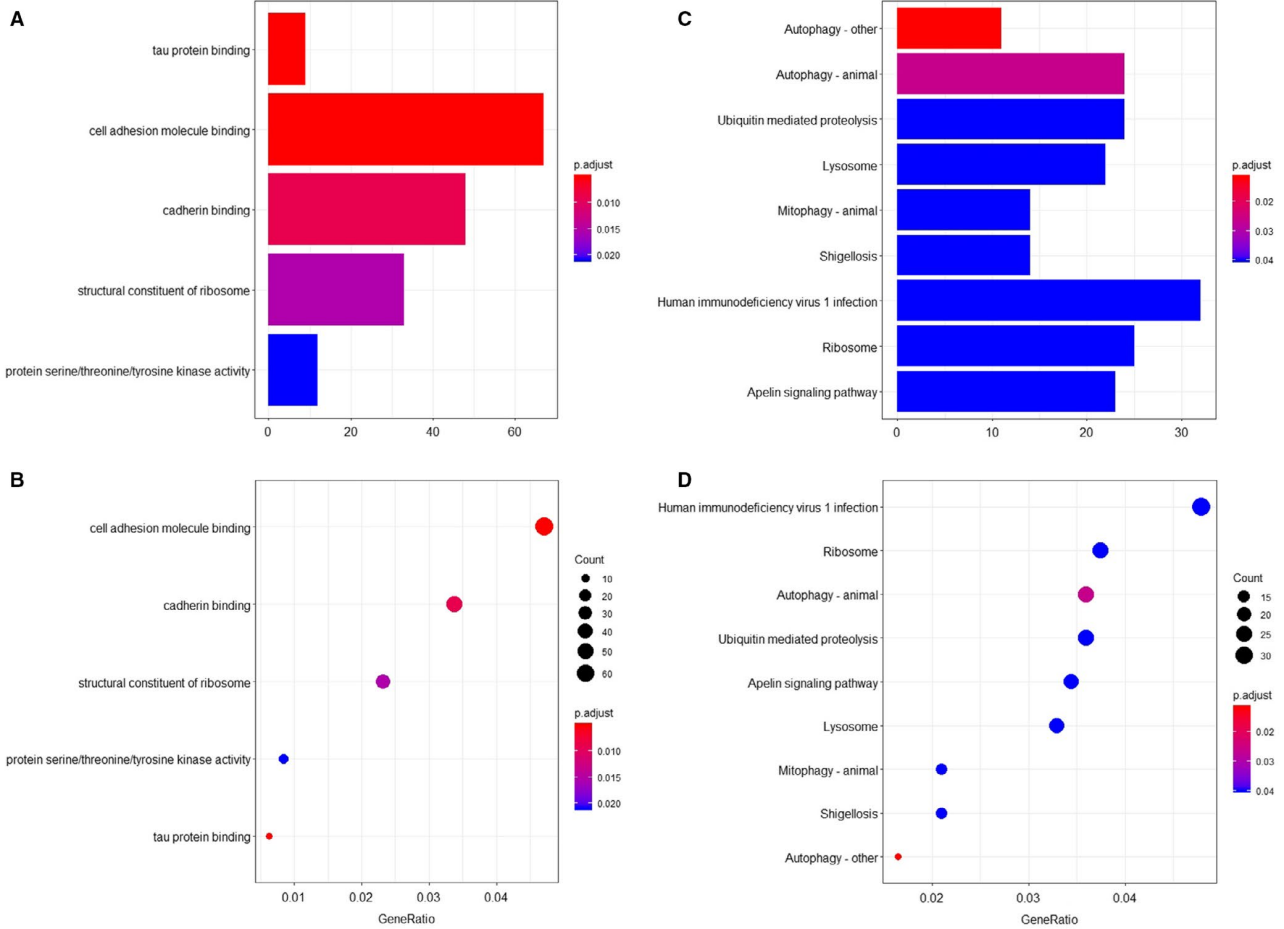
The enrichment analysis of gene symbols in survival‐associated alternative splicing events. The top 20 enriched terms are displayed. A, A bar chart showing the terms of molecular function. B, A bubble chart showing the terms of molecular function. C, A bar chart showing the Kyoto Encyclopedia of Genes and Genomes (KEGG) pathways. D, A bubble chart showing the KEGG pathways

**Figure 5:**
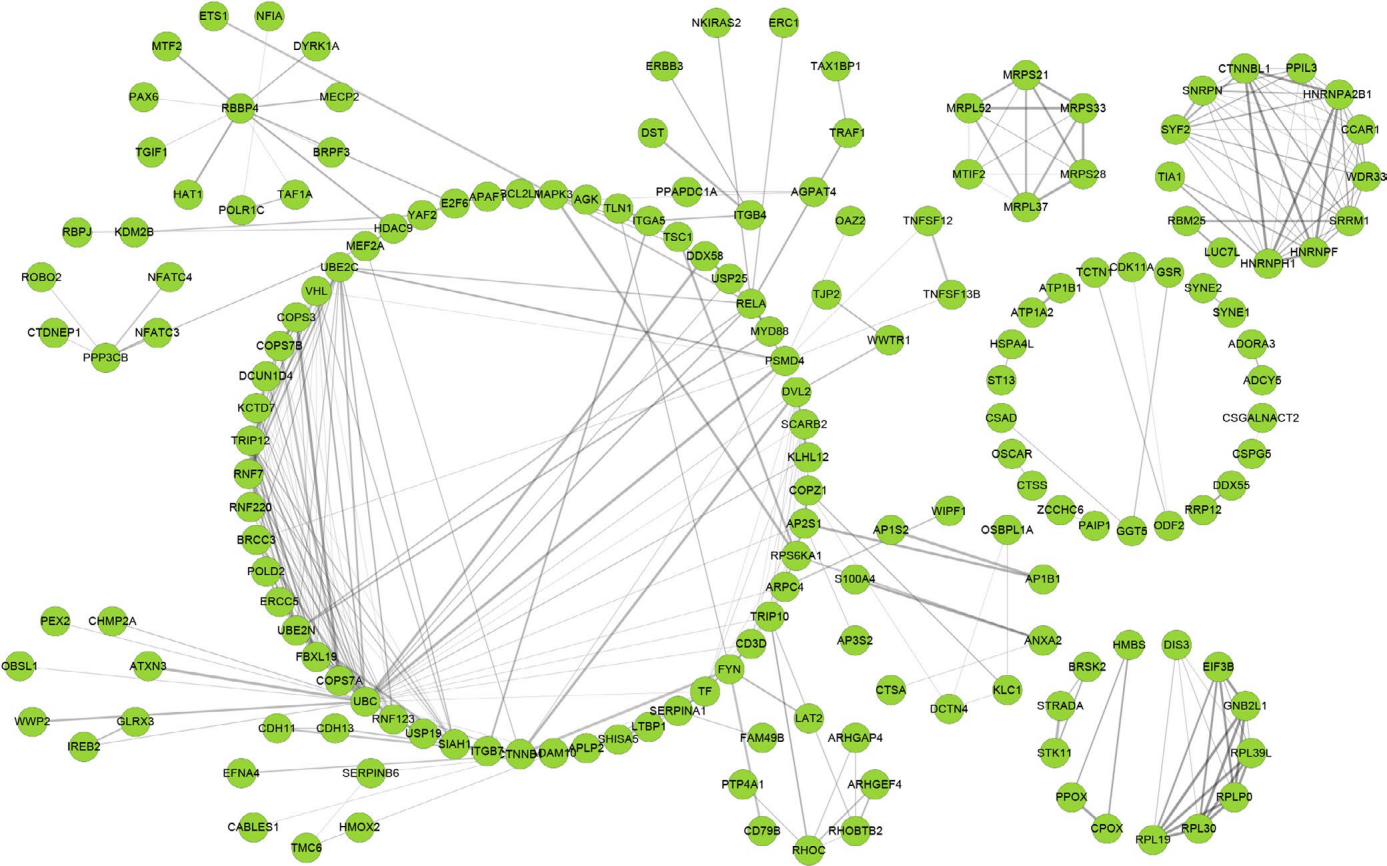
The protein‐protein interactive network of gene symbols in survival‐associated alternative splicing events. Each node represents a protein coded by a gene. The edges represent the interactions, among which the thicker edges indicate a higher combined score, while the darker edges indicate a higher coexpression

The ClusterProfiles R package was used to conduct enrichment analysis. Gene ontology enrichment consists of three categories: biological process, cellular component, and molecular function. In biological process, the top three enriched terms were establishment of protein localization to membrane, protein targeting to membrane, and vacuole organization (Figure 3A,B). In cellular component, the top significant terms were focal adhesion, cell‐substrate adherens junction, and cell‐substrate junction (Figure 3C,D). In molecular function, tau protein binding, cell adhesion molecule binding and cadherin binding were the first three significantly enriched terms (Figure 4A,B). A Kyoto Encyclopedia of Genes and Genomes analysis revealed some important pathways, such as autophagy pathway, ubiquitin‐mediated proteolysis, lysosome pathway, and apelin signaling pathway (Figure 4C,D). Taken together, the AS genes mainly enriched in autophagy related processes. Autophagy has been widely reported in biology and pathogenesis of GBM.^39^, ^40^, ^41^, ^42^ These may indicate that splicing of some core genes in autophagy may be important in GBM. In Figure 5, the protein interactive network presents the core genes, like UBC, VHL, KCTD7, FBXL19, RNF7, and UBE2N. These core genes may play an important role in GBM, which is worthy of more focus. For example, VHL inactivation by ID2 protein has been discovered to be a mechanism of inhibition of GBM growth.^43^

### 3.4 Prognostic signatures of AS events

**Figure 6:**
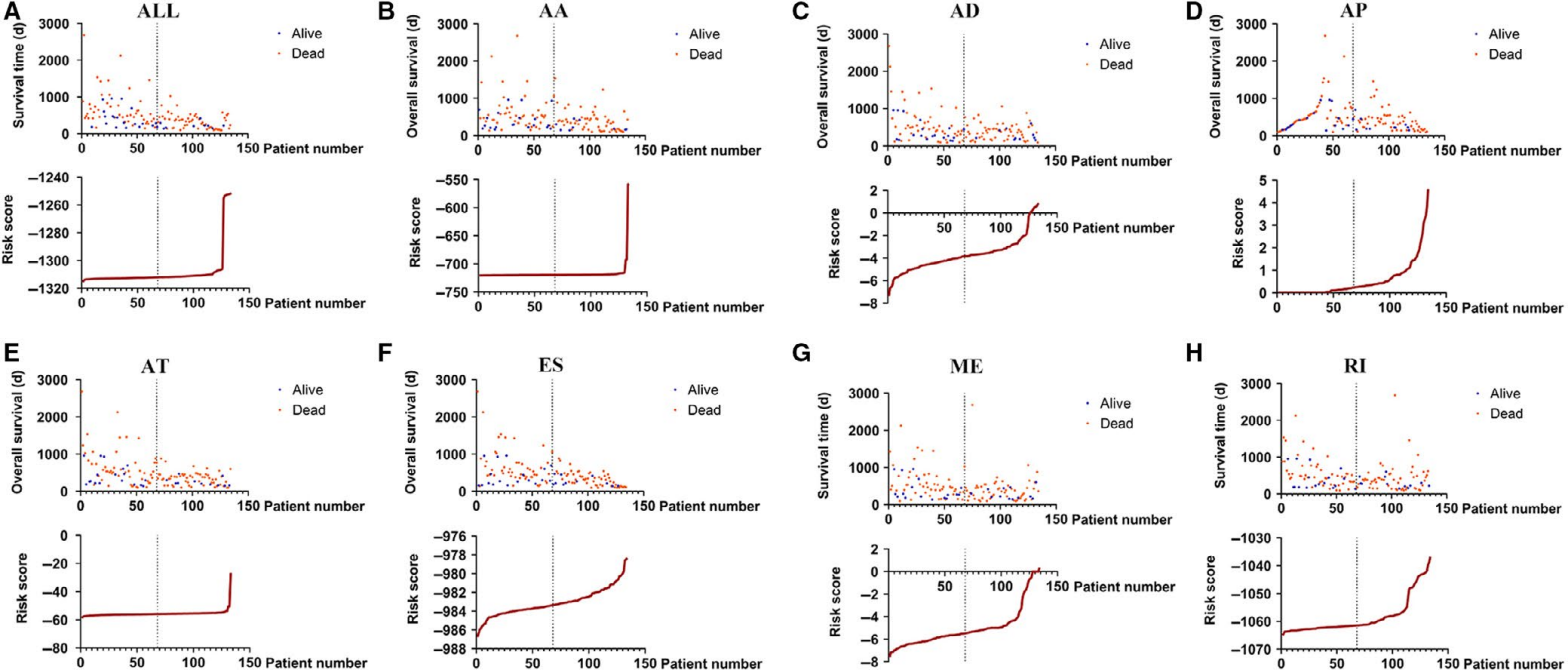
The survival details of the included patients in each prognostic model. The top of each drawing shows the survival data divided into low‐/high‐risk groups according to the median value of the risk score. The bottom shows the curve of the risk score. A‐H, The seven combined alternative splicing models, Alternate Acceptor site model, Alternate Donor site model, Alternate Promoter model, Alternate Terminator model, Exon Skip model, Mutually Exclusive exons model, and Retained Intron model

**Figure 7:**
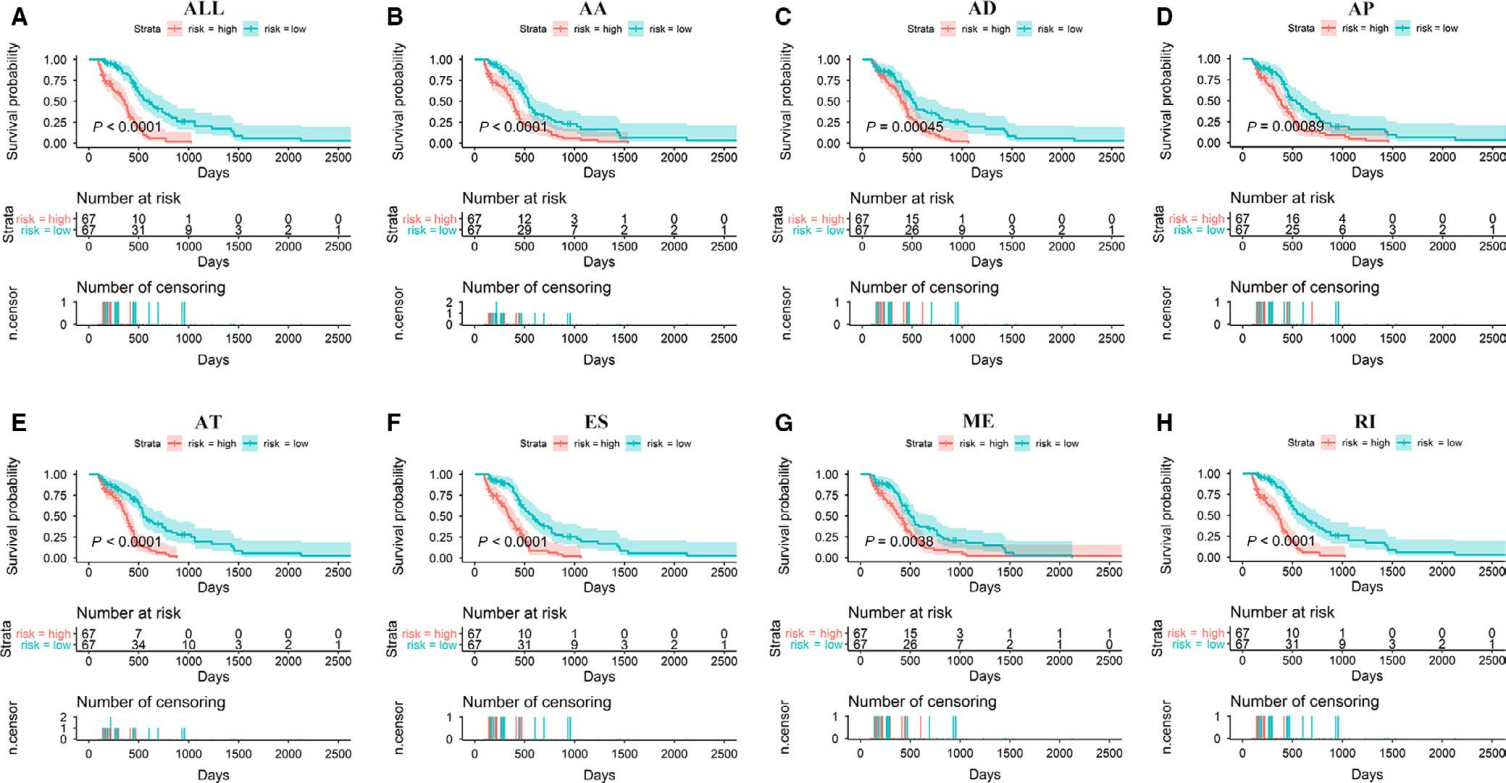
The survival curve of glioblastoma patients. The patients were divided into low‐/high‐risk groups according to the median value of their risk score. A *P* value below .05 was regarded as statistically significant. A‐H, The seven combined alternative splicing models, Alternate Acceptor site model, Alternate Donor site model, Alternate Promoter model, Alternate Terminator model, Exon Skip model, Mutually Exclusive exons model, and Retained Intron model

**Figure 8:**
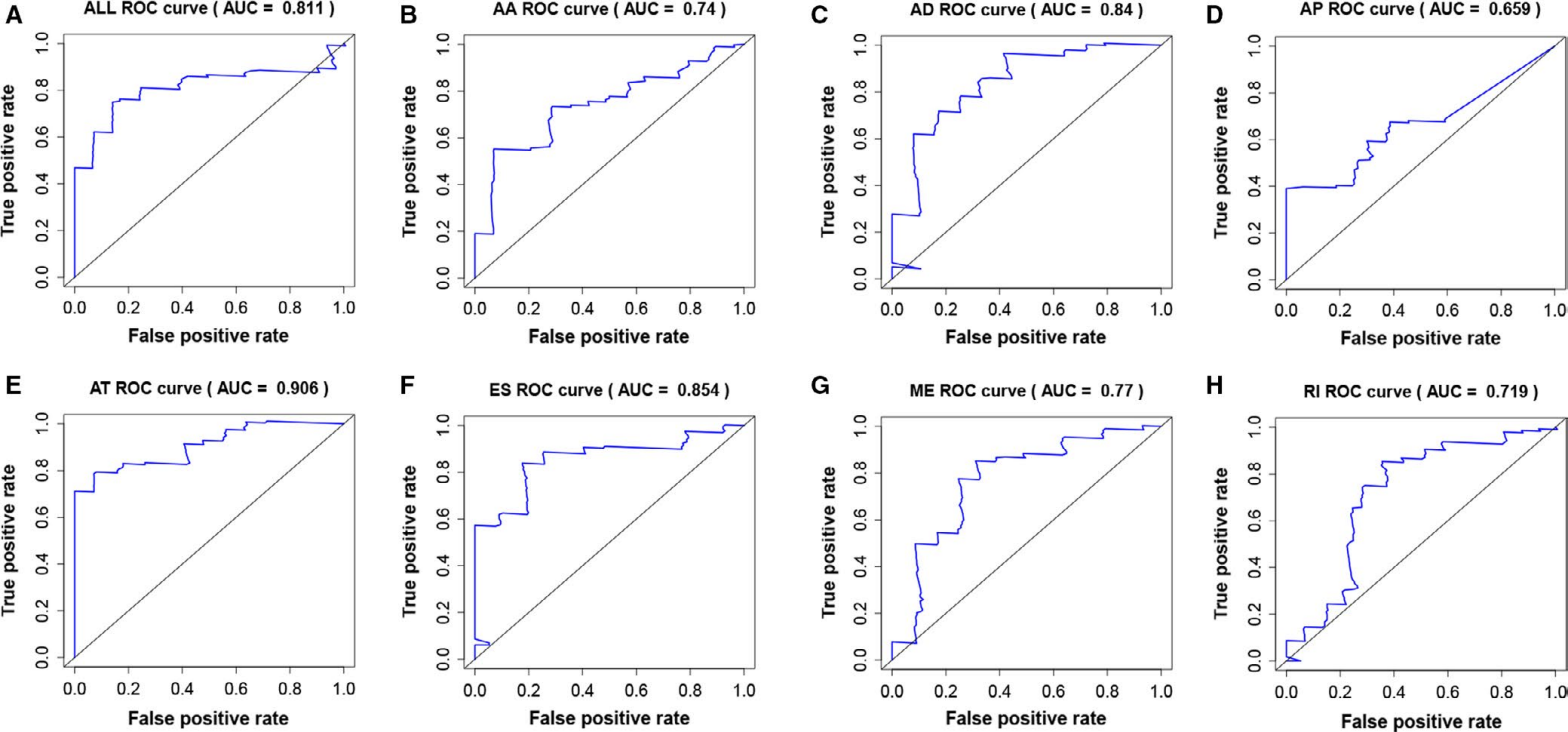
The receiver operating characteristic curve of each prognostic model. A‐H, The seven combined alternative splicing models, Alternate Acceptor site model, Alternate Donor site model, Alternate Promoter model, Alternate Terminator model, Exon Skip model, Mutually Exclusive exons model, and Retained Intron model

Independent prognostic AS events for each type of splicing were utilized to construct the prognostic predictors. The survival data of the included patients are displayed in Figure 6. Detailed information on the prognostic AS events revealed by the multivariate Cox regression is shown in Table 1. Figure 7 indicates that GBM patient survival in the high‐risk group was significantly poorer than in the low‐risk score group for all prognostic signatures. These indicated that more distinct molecular characteristics of AS events are adverse prognostic factors for GBM patients. To compare the predictive efficacy of each prognostic model, we performed survival ROC analysis. As displayed in Figure 8, the AUC of AA, AD, AP, AT, ES, ME, RI, and the seven combined‐AS models was 0.740, 0.840, 0.659, 0.906, 0.854, 0.770, 0.719, and 0.811, respectively. The prognostic model of AT showed the best predictive performance, followed by the ES model. There is significant potential for the AT splicing model to predict the survival of GBM patients.

**Table 1:** Information of AS events in each prognostic signature

Type	ID	B	HR	Lower	Upper	*P*
AA	EFNA4_AA_7933	−1.611	0.2	0.081	0.494	<.001
	GTDC1_AA_55511	0.85	2.339	1.645	3.326	<.001
	POLD2_AA_79475	0.792	2.208	1.329	3.668	.002
	RELA_AA_16902	−6.099	0.002	0	0.142	.004
	RPS6KA1_AA_1286	−0.269	0.764	0.657	0.889	<.001
AD	ANXA2_AD_30958	1.338	3.812	1.485	9.787	.005
	CHTF18_AD_33022	−0.094	0.911	0.869	0.954	<.001
	TF_AD_66850	2.019	7.528	2.336	24.26	.001
	ZBTB45_AD_52479	0.047	1.048	1.023	1.073	<.001
	ZNF302_AD_48995	−0.065	0.937	0.902	0.974	.001
AP	ADORA3_AP_4173	3.497	33.008	4.532	240.425	.001
	AP1B1_AP_61602	3.132	22.93	2.467	213.151	.006
	HDAC9_AP_78886	0.019	1.019	1.007	1.032	.002
	KLK10_AP_51263	0.082	1.086	1.023	1.153	.007
	PPAPDC1A_AP_13280	0.085	1.089	1.02	1.162	.011
AT	BTNL9_AT_75038	−0.085	0.919	0.87	0.97	.002
	COX6B2_AT_52075	−0.279	0.757	0.663	0.864	<.001
	CSGALNACT2_AT_11317	−0.241	0.786	0.674	0.917	.002
	DST_AT_76557	−0.177	0.838	0.714	0.984	0.031
	SYNE1_AT_78181	−0.103	0.902	0.863	0.943	<.001
ES	CCAR1_ES_11956	0.133	1.142	1.051	1.241	.002
	PPP3CB_ES_12157	0.518	1.679	1.058	2.665	.028
	TRIT1_ES_1916	−2.407	0.09	0.015	0.531	.008
	WWOX_ES_37677	−2.111	0.121	0.056	0.263	<.001
	DMTF1_ES_80295	−1.467	0.231	0.093	0.57	.001
	HAT1_ES_55964	−0.265	0.768	0.659	0.894	.001
	PPA2_ES_70200	3.831	46.096	7.177	296.055	<.001
	ST13_ES_62398	−3.728	0.024	0.004	0.134	<.001
ME	C4orf29_ME_70560	−0.05	0.951	0.91	0.994	.025
	OPN3_ME_204971	0.151	1.163	1.023	1.322	.021
	RPE_ME_100824	−0.02	0.98	0.967	0.993	.002
	SRPK1_ME_75933	−0.289	0.749	0.582	0.964	.025
	TGFBI_ME_73474	19.844	4.15E + 08	1710.923	1.01E + 14	.002
RI	ARMCX4_RI_89653	−0.039	0.961	0.927	0.997	.033
	HYAL1_RI_64995	−0.149	0.861	0.795	0.933	<.001
	LUC7L_RI_32846	−0.024	0.976	0.954	0.999	.037
	MS4A6A_RI_16057	−0.08	0.923	0.862	0.988	.021
	RPL30_RI_84641	0.457	1.579	1.066	2.339	.023
	SLC45A4_RI_85333	−0.058	0.943	0.919	0.968	<.001
	TMOD3_RI_30632	−3.267	0.038	0.007	0.202	<.001
	UBAP2_RI_86134	−7.086	0.001	0	0.035	<.001
ALL	RRP12_ES_12699	−0.6	0.549	0.433	0.695	<.001
	ST13_ES_62398	−2.793	0.061	0.008	0.444	.006
	UBAP2_RI_86134	−5.526	0.004	0	0.071	<.001
	SYNE1_AT_78181	−0.114	0.892	0.848	0.938	<.001
	HSD11B1L_ES_46873	0.041	1.041	1.018	1.066	.001
	TRIT1_ES_1916	−3.337	0.036	0.009	0.143	<.001
	KLC1_AD_29492	−0.786	0.456	0.302	0.687	<.001

Abbreviations: AA, alternate acceptor site; AD, alternate donor site; AP, alternate promoter; AT, alternate terminator; ES, exon skip; ME, mutually exclusive exons; RI, retained intron, HR, hazard ratio.

### 3.5 Differentially expressed splicing factors and splicing network

**Figure 9:**
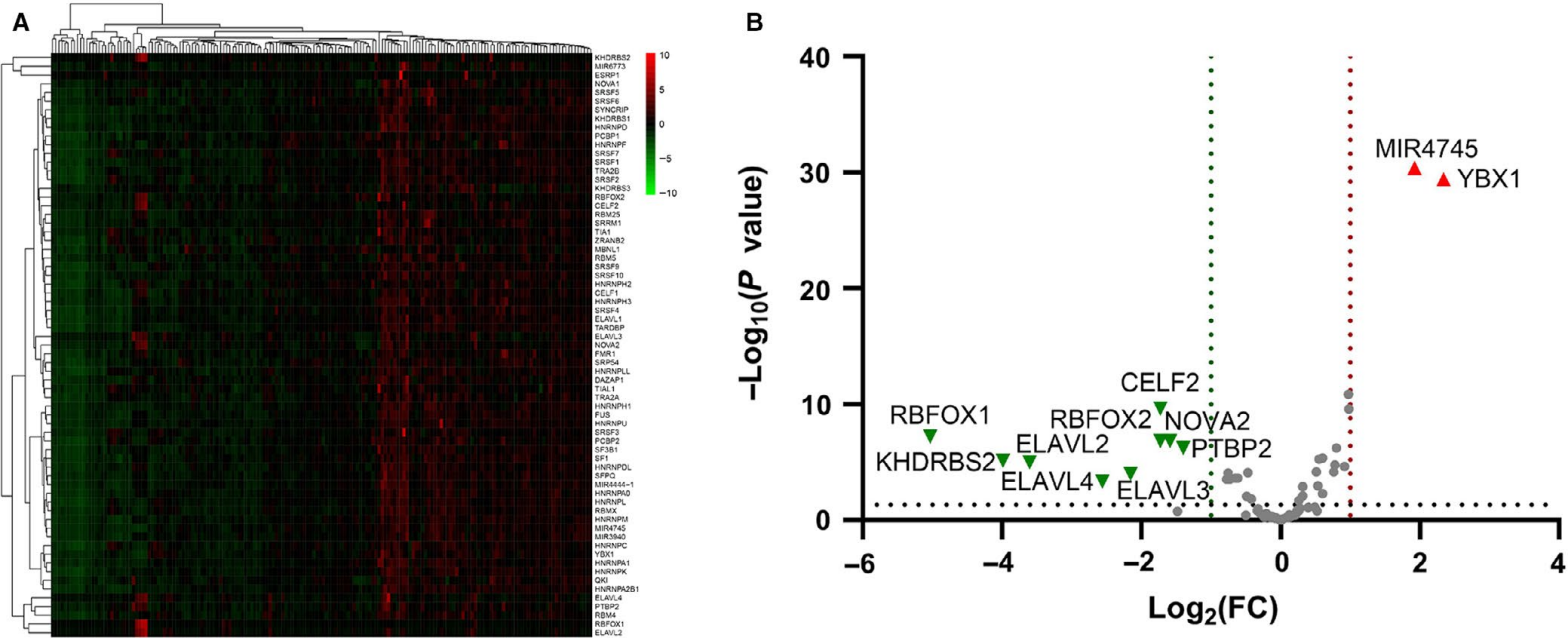
The Differentially expressed splicing factor analysis. A, A heat map displaying the expression of each splicing factor. B, A volcano plot displaying the significantly differentially expressed splicing factor. The red triangles represent the up‐regulated splicing factors (fold change > 2; *P* < .05), while the green triangles represent the down‐regulated splicing factors (fold change < 0.5; *P* < .05)

**Figure 10:**
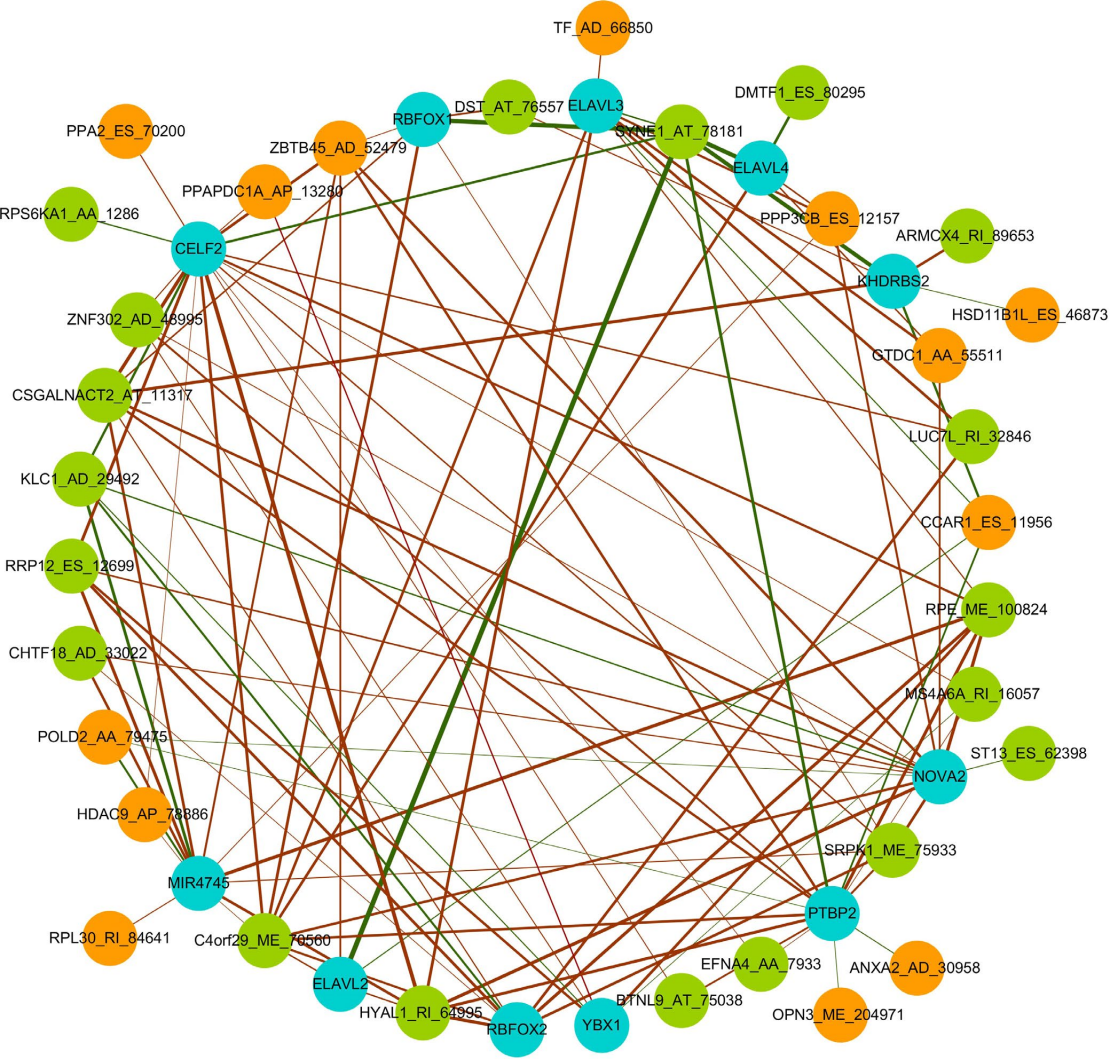
The splicing regulatory network constructed using splicing factors and alternative splicing factors. The blue nodes represent differentially expressed splicing factors. The orange nodes represent the unfavorable alternative splicing events (Hazard ratio > 1), while the green nodes represent favorable alternative splicing events (Hazard ratio < 1). The red edges represent the positive correlations, while the green edges represent negative correlations. The thicker edges indicate stronger correlations

**Figure 11:**
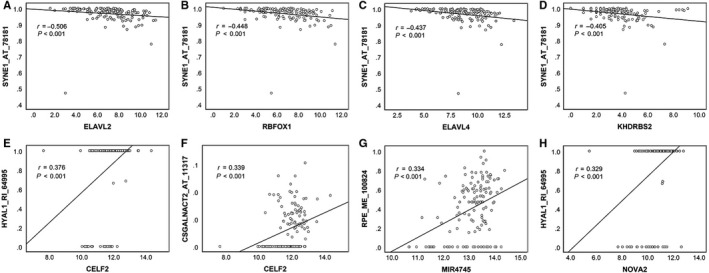
A linear correlation plot displaying the strongest correlations between splicing factors and alternative splicing events. A, ELAVL2 was negatively correlated to SYNE1_AT_78181. B, RBFOX1 was negatively correlated to SYNE1_AT_78181. C, ELAVL4 was negatively correlated to SYNE1_AT_78181. D, KHDRBS2 was negatively correlated to SYNE1_AT_78181. E, CELF2 was positively correlated to HYAL1_RI_64995. F, CELF2 was positively correlated to CSGALNACT2_AT_11317. G, MIR4745 was positively correlated to RPE_ME_100824. H, NOVA2 was positively correlated to HYAL1_RI_64995

A total of 67 splicing factors were gathered during the differential expression analysis. The expression of splicing factors in each GBM sample and normal tissue was visualized as a heatmap (Figure 9A). Eleven significantly differentially expressed splicing factors were identified. Two (MIR4745 and YBX1) were up‐regulated, and nine (RBFOX1, RBFOX2, ELAVL2, ELAVL3, ELAVL4, KHDRBS2, CELF2, NOVA2, and PTBP2) were down‐regulated (Figure 9B). Regarding the prognostic value of the splicing factors in GBM, no statistical significance was observed in the survival analysis (data not shown). Spearman correlation analysis was performed to investigate the correlation between differentially expressed splicing factors and independent prognostic AS events. A total of 106 correlations, including 26 negative correlations and 80 positive correlations were identified. The positive regulations between splicing factors and AS events occurred more frequently than negative regulations. The correlation network was constructed to display the regulatory relationship (Figure 10). The top correlations were displayed via linear correlation plots (Figure 11).

## 4 DISCUSSION

In this study, the entire prognostic AS events in GBM were identified using the TCGA splicing profile for the first time. Using the splicing prognostic signatures to predict the survival of GBM patients shows excellent potential for clinical practice. Splicing network constructed using AS events and splicing factors lays the foundation for future research on the splicing regulatory mechanism in GBM. The survival‐associated splicing in this study shows promise for novel targeted therapy in GBM.

AS is a universal and pivotal mechanism in pre‐mRNA processing, allowing for considerable proteomic diversity and complexity in a relatively limited human genome.^44^ Research efforts over the last few decades have increased our knowledge on the alteration of AS in diseases, including cancer. The aberration of AS in cancers may present in various ways, which makes the mechanism sophisticated and obscure.^45^ The aberrant splicing of pre‐mRNA contributes to various cell functions, such as proliferation, invasion, migration, metastasis, apoptosis, and drug resistance.^46^ For example, AS of TCF‐4 was found to inhibit the proliferation and metastasis of lung cancer cells.^47^ Chen et al found that CD44 splicing was associated with invasion and migration in ovarian cancer.^48^ The splicing variants of TP53, FAS, CASP9, and BCL2L1 have been associated with cancer cell apoptosis and survival.^18^, ^49^ Calabretta's study showed that modulating the pyruvate kinase gene (PKM) splicing could promote gemcitabine resistance in pancreatic cancer cells.^50^ Recently, studies have focused on investigating the potential therapeutic value of AS in cancers. Scholars have found that splicing pre‐mRNA, such as human telomerase reverse transcriptase (hTERT) and epidermal growth factor receptor (EGFR), is correlated to cancer progression and prognosis.^51^, ^52^ Some researchers have begun to link AS to cancer subtypes, exploring their influence on prognosis. For example, Leivonen et al utilized AS to discriminate between the molecular subtypes and determine the prognostic impact in diffuse large B‐cell lymphoma.^53^ In 2017, Li et al pioneered an investigation of prognostic AS events to construct prognostic signatures for predicting the survival of non‐small cell lung cancer patients using TCGA data.^25^ Similar approaches were used to research other tumors, such as bladder cancer,^26^ prostate cancer,^27^ ovarian cancer,^28^ esophageal carcinoma,^54^ and gastrointestinal adenocarcinomas.^29^ These studies have applied AS to clinical practice regarding cancer, providing predictive references for prognosis at a molecular level. However, no such study has been carried out for clinical practice regarding GBM. We conducted this study to provide survival‐associated AS events and construct prognostic models for predicting the survival of GBM patients. Moreover, enrichment analysis, protein network, and splicing network were performed to investigate the potential molecular regulatory mechanism of AS in GBM.

In this study, 2355 survival‐associated AS events in GBM were identified. Each independent survival‐associated AS event, which could be a potential therapy target, is worth being focused in GBM‐related research. Here, we focused on their combined effect in survival prediction of GBM patients. Prognostic models constructed using each type of AS event showed that a high risk of AS signatures indicated worse survival rates. When applying the prognostic models to clinical practice, it is especially important to adopt more active therapeutic strategies for those patients with high risk of AS events. The best predictive model was the AT model, showing a predictive AUC at 0.907. Previous studies have applied miRNA and mRNA to the construction of prognostic signatures for predicting the survival of GBM patients. In Xiong's study, the predictive AUC of integrated RNA, mRNA‐only, and miRNA‐only signatures were 0.828, 0.742, and 0.757, respectively.^55^ Our prognostic model of AT splicing showed better predictive performance than the mRNA and miRNA models. Therefore, applying AS prognostic signatures to predict a GBM patient's survival is promising.

We wondered what pathways the gene symbols of prognostic AS events would enrich. Therefore, we performed enrichment analysis and revealed important pathways. The autophagy‐related processes were the most enriched regarding GBM. Autophagy is an intracellular degradative process, which exerts pivotal functions in maintaining metabolism and homeostasis.^56^, ^57^, ^58^ In cancer biology, autophagy may exert dual effects in tumor promotion and suppression.^59^, ^60^, ^61^, ^62^ Targeting autophagy has been a focus in searching for novel therapies for cancers.^63^, ^64^, ^65^, ^66^ With GBM, scholars have previously noted the significant role of autophagy. For example, Peng et al discovered that TRIM28 could activate autophagy, thus promoting cell proliferation in GBM.^67^ Regulating autophagy was the key process involved in the chemoresistance and radioresistance of GBM.^42^ Sensitizing GBM cells to temozolomide via regulating autophagy has been widely reported by scholars.^68^, ^69^, ^70^, ^71^, ^72^ With respect to the interplay between autophagy and AS, splicing autophagy‐related genes has been reported to influence autophagy.^73^, ^74^ However, no study has reported the correlation between AS and autophagy in GBM. We speculated that the role of autophagy and its correlation with AS might be an interesting molecular mechanism in GBM worthy of future focus.

The spliceosome, consisting of five small nuclear RNAs (U1, U2, U4, U5, and U6) and abundant protein factors, is the place where AS happens.^75^ Splicing factors are a core protein in the spliceosome, playing pivotal roles in regulating the splicing process. Two well‐studied splicing factor families, the serine‐rich proteins and the heterogenous nuclear ribonucleoproteins, have been extensively reported in cancers.^76^, ^77^ In our study, we constructed an interesting splicing network to illuminate the potential splicing regulatory mechanism. Positive correlations were more common than negative correlations between splicing factors and AS events in GBM. A single splicing factor may play dual roles in the positive and negative regulation of different AS events. For a single AS event, different splicing factors generally exert a synergistic effect, but there are exceptions. For instance, MS4A6A_RI_16057 was negatively regulated by YBX1, while it was positively regulated by PTBP2 and CELF2. These suggested a complex regulatory mechanism between splicing factors and AS events. The results provided a better understanding of the GBM spliceosome.

Several issues that arose in this study must be addressed. First, the study was based solely on online database sources. There is no cross validation for the results, which is certainly a limitation in this study. It is necessary to validate the results using other datasets and experiment in the future. Second, the survival analysis method in this study is based on Cox regression. However, in Shen's study,^78^ a statistical method named SURVIV (Survival analysis of mRNA Isoform Variation) was reported, which is designed for analyzing mRNA isoform and patients' survival. The authors of the study suggested that SURVIV outperforms the conventional Cox regression survival analysis. Therefore, the statistical methods used in this study can be improved in future research. Besides, though we discovered that autophagy is an important process in GBM, its relationship with AS remains unclear. Thus, this requires further research. Additionally, although we showed the regulatory function of the splicing factors in AS, the effect of other regulatory factors remains unknown. The prognostic value of splicing factors in GBM did not show statistical significance. More studies to elucidate the comprehensive splicing regulatory network and the prognostic value of regulatory factors are necessary.

In short, we have established data concerning the entirety of survival‐associated AS events in GBM. The prognostic signatures constructed using AS events were also found to show satisfactory predictive efficacy for the survival of GBM patients. The splicing regulatory network between the splicing factors and AS events provided a better understanding of the GBM spliceosome. The work achieved in this study underpins future splicing research and provides novel perspectives regarding potential GBM therapy.

## CONFLICT OF INTEREST

None declared.

## Data Availability

All the data used in the manuscript are freely available online.
